# RELATIONSHIPS MATTERS: INTERGENERATIONAL CONFLICT PREDICTS STRESS AND DEPRESSION IN GRANDPARENT CAREGIVERS

**DOI:** 10.1093/geroni/igad104.0401

**Published:** 2023-12-21

**Authors:** Loriena Yancura

**Affiliations:** University of Hawa`i at Manoa, Honolulu, Hawaii, United States

## Abstract

Literature from both research and applied perspectives suggest that studies consider aspects of grandparent involvement beyond residence and custodial status to examine predictors and correlates of grandparent well-being. These suggestions are supported by a multigenerational family life course framework, which considers lifelong linkages among family and notes that interactions among family members are critically important for well-being. Objective: This study hypothesized that stress and burden in caregiving grandparents (G1) would be predicted by relationship quality with the grandchildrens’ parents (G2) when other variables are controlled. Method: Data for these analyses were collected online via Qualtrics Panel Service. Surveys were completed by 312 grandparents who provided care to their grandchildren for at least 20 hours per week.

Results: After controlling for grandchild age and grandparent household status (non-resident, skipped generation, multigeneration), stress (R2=.21) and depression (R2= .34) were positively predicted by non-White race, lower income, and grandparent age. Adding grandchild behavior and grandparent relationship with G2 significantly increased predictive power (p<.001) for both stress (R2=.51) and ( R2=.41) depression.

Discussion: These findings suggest that variables beyond residence status are important in understanding the effect of providing care to grandchildren on grandparents’ well-being.

